# Genome-wide SNP analysis uncovers population structure and genetic differentiation in longan (*Dimocarpus longan* Lour.)

**DOI:** 10.3389/fpls.2026.1796047

**Published:** 2026-04-07

**Authors:** Hong-Ye Qiu, Xian-Quan Qin, Chen Fang, Yan-Jie Hou, Dong-Bo Li, Jing-Yi You, Ning Xu, Xiao-Lin Cai, Hongli Li

**Affiliations:** Horticulture Research Institute, Guangxi Academy of Agricultural Sciences, Nanning, China

**Keywords:** *Dimocarpus longan*, genetic diversity, germplasm resources, population genomics, population structure

## Abstract

Longan (*Dimocarpus longan* Lour.) is an economically important fruit tree in tropical and subtropical Asia, yet population-level genomic resources supporting germplasm conservation and breeding remain limited. We resequenced 185 longan accessions and generated a high-density genome-wide SNP dataset for population genomic analyses, including phylogenetic inference, principal component analysis, model-based ancestry estimation, linkage disequilibrium analysis, and genome-wide diversity/differentiation scans. The analyses consistently resolved the panel into four genetic groups, while several accessions showed mixed ancestry, suggesting historical germplasm exchange. Genome-wide diversity and differentiation analyses indicated heterogeneous within-group variation but overall low differentiation among groups, with comparatively greater divergence involving group 4. Linkage disequilibrium decay revealed differences in haplotype structure among groups. A genome-wide scan integrating diversity reduction and elevated differentiation identified a narrow candidate interval on chromosome 11 (17.79–17.98 Mb) showing a localized sweep-like signature. These results provide a population-genomic framework for longan germplasm classification, conservation, and breeding-oriented follow-up.

## 1 Introduction

Longan (Dimocarpus longan Lour.) is an important perennial fruit tree cultivated widely across tropical and subtropical Asia, with major production and cultivar diversification in southern China and Southeast Asia. As in many woody perennials, longan improvement is constrained by long juvenility, high heterozygosity, and incomplete pedigree records, while extensive cultivar movement and clonal propagation can blur geographic signatures and complicate germplasm utilization (Iwata et al., 2016; Kerr et al., 2024; Khan et al., 2024; Pacheco-Ruiz et al., 2025).

Genomic resources for longan have advanced rapidly over the last decade, enabling whole-genome variant discovery and improved evolutionary and functional inference. A first draft genome provided foundational gene models and early diversity insights (Lin et al., 2017), and a chromosome-level assembly further improved contiguity and supported population genomic analyses with broader evolutionary resolution (Wang et al., 2022). More recently, an improved high-quality longan genome enhanced assembly completeness and facilitated refined analyses relevant to flowering and adaptation (Zhao et al., 2026). Despite these genomic advances, what remains limited is a population-level framework that jointly interprets ancestry structure, relatedness, LD patterns, and diversity–differentiation landscapes within a single germplasm panel and relates these features to germplasm management and breeding-oriented prioritization.

In Sapindaceae and other perennial fruit crops, domestication and improvement histories are often non-linear, shaped by repeated introductions, region-specific selection, and introgression. A close relative, lychee, provides a compelling example in which genome-enabled analyses revealed structured diversity and evidence consistent with complex domestication and subsequent crosses rather than a simple bifurcating history (Hu et al., 2022). More broadly, recent syntheses emphasize that translating genomics into breeding gains in perennial systems requires integrating population structure and germplasm exchange histories with genomic resources, including explicit attention to wild relatives and introgression for adaptation and resilience (Migicovsky, 2025; Kerr et al., 2024; Alemu et al., 2024).

Genome scans based on diversity depletion and elevated differentiation can prioritize candidate intervals potentially shaped by long-term selection, although such signals may also reflect demography or background selection (Weir and Cockerham, 1984; Danecek et al., 2011). In many plant population-genomic studies, biological interpretation can be strengthened by linking outlier intervals to annotated gene content and pathway-level hypotheses when coordinate-consistent annotation resources are available (Yu et al., 2012; Raudvere et al., 2019; Kolberg et al., 2020). In the present study, however, we use genome-wide sweep-like signals conservatively to prioritize candidate intervals for future follow-up rather than to claim resolved gene-level mechanisms.

Here, we performed whole-genome resequencing of 185 longan accessions and generated a dense SNP resource to address three biological questions. First, we asked how longan germplasm is structured at the genome-wide level and whether the inferred groups are broadly consistent with sample origin and possible historical germplasm movement. Second, we evaluated whether these groups differ in genome-wide diversity, differentiation, and LD patterns in ways consistent with distinct demographic and improvement histories. Third, we used integrated diversity- and differentiation-based genome scans to prioritize genomic intervals showing localized signatures consistent with long-term selection. Because admixture-graph inference can be sensitive to model assumptions, TreeMix-derived gene-flow signals were interpreted conservatively and considered together with independent evidence from structure and differentiation analyses. Together, this study provides a population-genomic baseline for longan germplasm classification, conservation, and breeding-oriented follow-up.

## 2 Materials and methods

### 2.1 Plant materials and whole-genome resequencing

A total of 185 Dimocarpus longan Lour. accessions were included in this study. These accessions were collected from a curated germplasm panel maintained by the Horticulture Research Institute, Guangxi Academy of Agricultural Sciences, and represent major longan-producing regions in China together with several accessions from Southeast Asia. Detailed metadata for each accession, including accession name, province or country of origin, and germplasm source information, are provided in **Supplementary Table 1**. Young, healthy leaves were collected from each accession and immediately frozen in liquid nitrogen prior to DNA extraction. Genomic DNA was extracted using a modified CTAB protocol optimized for polyphenol-rich perennial leaf tissues. Briefly, polyvinylpyrrolidone (PVP) was added to the extraction buffer to reduce interference from polyphenolic compounds, RNA was removed by RNase A treatment, and an additional chloroform:isoamyl alcohol extraction step was included to improve DNA purity. DNA purity was assessed using a NanoDrop spectrophotometer based on A260/280 and A260/230 ratios, DNA concentration was quantified using a Qubit fluorometer with the dsDNA HS assay, and DNA integrity was evaluated by 1% agarose gel electrophoresis.

Paired-end libraries (2 × 150 bp) were prepared following the manufacturer’s standard protocol and sequenced on an Illumina NovaSeq 6000 platform to generate whole-genome resequencing data with an average depth of approximately 10× per accession. Raw read quality metrics, including GC content, Q20, and Q30, were generated using FastQC and summarized with MultiQC (**Supplementary Table 1**).

### 2.2 Read preprocessing, mapping, and SNP calling

Raw paired-end reads were processed using fastp v0.23.2 to remove adapter contamination, trim low-quality bases, and discard reads shorter than 50 bp after trimming. Clean reads were aligned to the published longan reference genome of cultivar ‘Honghezi’ from Lin et al. (2017) using BWA-MEM v0.7.17 with default parameters unless otherwise stated. The reference FASTA used for read mapping was longan.scaffold.fa.gz from the GigaDB dataset associated with Lin et al. (2017).

SAM files were converted to BAM format, sorted, and indexed using SAMtools v1.15, and PCR duplicates were marked using Picard MarkDuplicates v2.27.1. Variants were called for all accessions using bcftools v1.15.1 in a cohort-based workflow, and raw variants were exported in VCF format. To generate a high-confidence SNP set for downstream population genomic analyses, we retained only biallelic SNPs and excluded indels. We further filtered the SNP dataset to remove loci with low confidence and then applied downstream population-genetic filters of minor allele frequency (MAF) > 0.05 and missing rate < 0.20 across the 185 accessions. The final filtered dataset was used for population structure, kinship, LD, diversity, differentiation, and selection-scan analyses.

### 2.3 Population structure analyses

Population structure was inferred using multiple complementary approaches. A neighbor-joining (NJ) phylogenetic tree was constructed based on genome-wide SNP-derived pairwise genetic distances. Principal component analysis (PCA) was performed to summarize major axes of genetic variation among accessions (Patterson et al., 2006).

Model-based ancestry inference was performed using ADMIXTURE (Alexander et al., 2009), with the number of ancestral populations (K) ranging from 1 to 10. To reduce the influence of linkage disequilibrium on ancestry inference, SNPs were LD-pruned prior to ADMIXTURE using a sliding-window approach implemented in PLINK (--indep-pairwise 50 5 0.2). The optimal K was selected based on the minimum cross-validation (CV) error, and ancestry proportions were visualized across accessions. The overall clustering pattern inferred by ADMIXTURE was interpreted together with the NJ tree and PCA results.

### 2.4 Kinship estimation and handling of closely related samples

Pairwise kinship among accessions was estimated from genome-wide SNPs using a genomic relationship matrix approach implemented in GCTA (Yang et al., 2011). Kinship coefficients were summarized to evaluate overall relatedness and to identify highly related pairs potentially reflecting clonal propagation, duplicated accessions/synonyms, or closely related materials. To assess robustness, we examined highly related pairs and conducted a sensitivity check by removing one accession from each high-kinship pair; the main population structure patterns were unchanged.

### 2.5 Inference of migration and gene flow

Historical gene flow among inferred genetic groups was investigated using TreeMix, which models population splits and migration events based on genome-wide allele frequency covariance (Pickrell and Pritchard, 2012). Group-level allele frequency inputs were constructed from the filtered SNP dataset. Migration edges were fitted on top of a maximum-likelihood population tree, and inferred gene-flow patterns were interpreted cautiously and cross-compared with population structure (PCA/ADMIXTURE) and differentiation summaries.

### 2.6 Genetic diversity and population differentiation

Within-group nucleotide diversity (π) and between-group population differentiation (FST) were calculated in sliding windows across the genome using VCFtools (Danecek et al., 2011). FST was estimated following the Weir–Cockerham framework (Weir and Cockerham, 1984). Negative FST values can occur due to sampling variance and were truncated to zero prior to summarization. Genome-wide mean π values were calculated for each group, and genome-wide mean FST values were calculated for each pairwise group comparison. Window-level π and FST results are provided in **Supplementary Table 2**.

### 2.7 Linkage disequilibrium analysis

Linkage disequilibrium (LD) was quantified using the squared correlation coefficient (r²) between SNP pairs in PLINK (Chang et al., 2015). Pairwise r² was computed within a maximum physical distance of 1,000 kb, and LD decay curves were generated by binning SNP pairs by physical distance and plotting mean r² against distance.

### 2.8 Detection of putative selection signals and candidate-interval follow-up

Genome-wide scans for putative selection were conducted by jointly considering windows with reduced nucleotide diversity (π) and elevated differentiation (FST) based on the sliding-window statistics (**Supplementary Table 2**). For each window, we derived composite scan metrics, including log2(πmax/πmin) across the four groups and FSTmax, defined as the maximum value among the six pairwise FST comparisons. Candidate windows were defined using stringent empirical thresholds (top 1% for both metrics), and adjacent candidate windows were merged into candidate intervals.

Candidate intervals were summarized as prioritized genomic regions for follow-up analyses. Gene-level annotation and functional enrichment of candidate intervals require a gene model set that is coordinate-consistent with the reference assembly used for SNP positioning. In this study, the available annotation resources from Lin et al. (2017) (GigaDB: 10.5524/100276; e.g., longan.gff.gz) are scaffold-based, and therefore cannot be used to unambiguously map candidate windows reported in chromosome coordinates to gene models without an appropriate scaffold-to-chromosome conversion. Accordingly, we report candidate intervals as genomic coordinates and treat gene annotation and GO/KEGG enrichment as downstream analyses to be performed once a coordinate-consistent annotation is available.

## 3 Results

### 3.1 Sequencing quality and genome-wide SNP discovery

Whole-genome resequencing of 185 longan accessions generated high-quality data for population genomic analyses (**Supplementary Table 1**). Sequencing depth was approximately 10× per accession, and base quality was consistently high across samples (mean Q20 = 97.58%, mean Q30 = 92.97%), with stable GC content (mean 36.49%) (**Supplementary Table 1**). After read mapping and variant calling, followed by stringent filtering (MAF > 0.05 and missing rate < 0.20), a total of 2,247,569 high-confidence SNPs were retained for downstream analyses. This genome-wide SNP dataset provides a dense marker resource for population structure inference, relatedness assessment, LD characterization, and genome-wide diversity/differentiation scans.

### 3.2 Population structure, ancestry composition, and relatedness of longan germplasm

**Figure 1:**
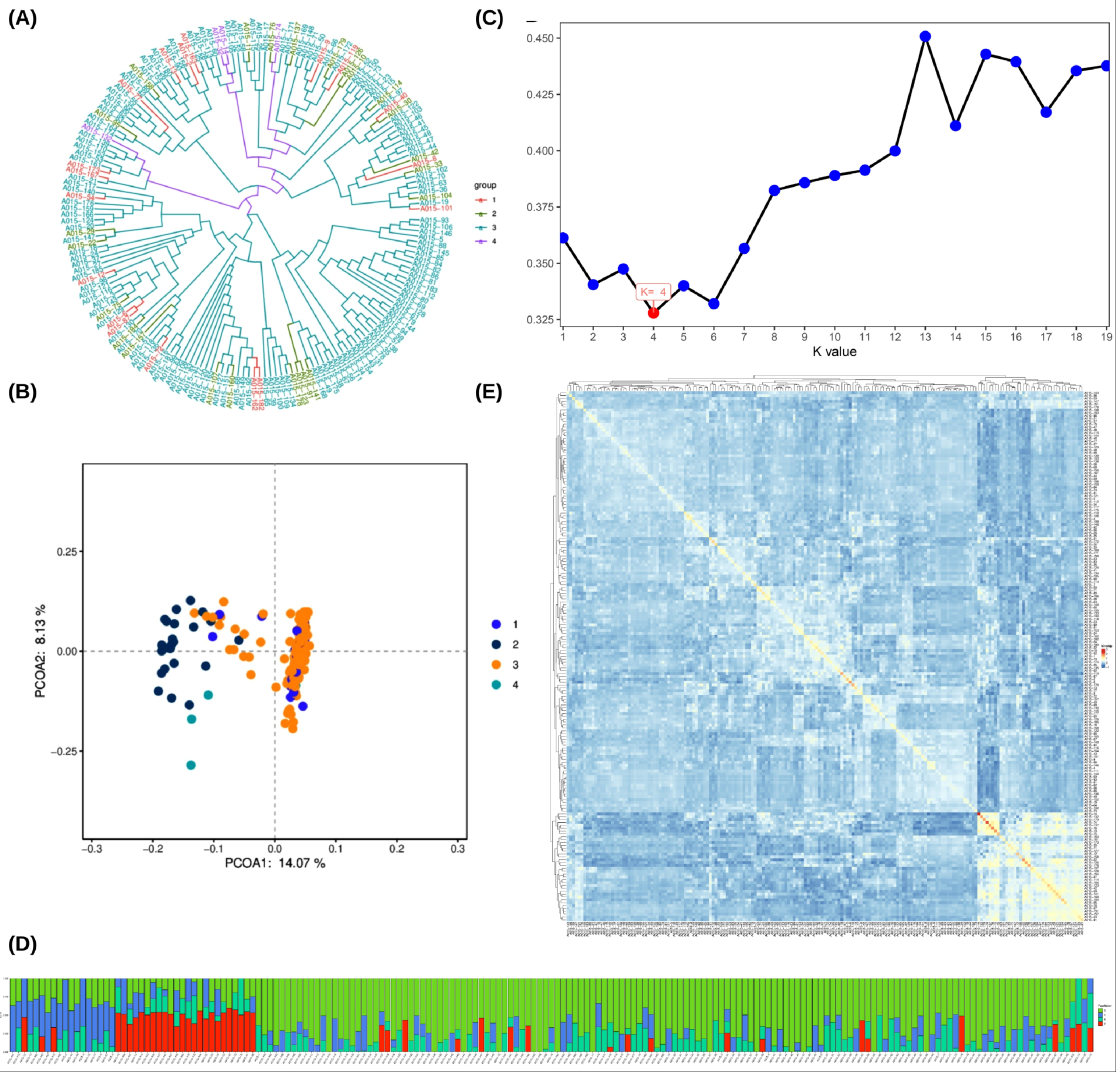
Population structure and genetic relationships among longan (Dimocarpus longan Lour.) accessions inferred from genome-wide SNPs. **(A)** Neighbor-joining (NJ) phylogenetic tree based on genome-wide SNPs, clustering the accessions into four genetic groups (Groups 1–4; colors correspond to group assignments). **(B)** Principal component analysis (PCA) of all accessions using genome-wide SNPs; the first two principal components (PC1 and PC2) are shown, and points are colored by the four inferred groups. **(C)** Cross-validation (CV) error across different numbers of ancestral populations **(K)** in ADMIXTURE; the minimum CV error supports K = 4. **(D)** ADMIXTURE ancestry proportions at K = 4; each vertical bar represents one accession, and the colored segments indicate the estimated membership coefficients for the four ancestral components. **(E)** Pairwise kinship heatmap calculated from genome-wide SNPs; warmer colors indicate higher relatedness and cooler colors indicate lower relatedness.

To resolve genetic structure within the resequenced panel, we performed phylogenetic reconstruction, principal component analysis (PCA), and ADMIXTURE inference based on genome-wide SNPs. The neighbor-joining phylogenetic tree consistently separated the longan panel into four genetic groups (group 1–4) (**Figure 1A**). PCA further supported this subdivision, with PC1, PC2, and PC3 explaining 14.07%, 8.13%, and 6.53% of total genetic variance, respectively (**Figure 1B**), indicating genome-wide structure among the four groups.

ADMIXTURE analysis identified K = 4 as the optimal number of ancestral clusters, supported by the minimum cross-validation error (CV error = 0.32791) (**Figure 1C**). Several accessions displayed mixed ancestry proportions (**Figure 1D**), consistent with historical admixture among groups. Kinship analysis indicated generally low pairwise relatedness across the panel: off-diagonal kinship coefficients were centered near zero (median −0.0196, IQR −0.1363 to 0.1062), with only a small fraction of pairs showing elevated relatedness (maximum 1.1471) (**Figure 1E**). We inspected highly related pairs and confirmed that the major PCA/ADMIXTURE clustering patterns remained unchanged after sensitivity checks, indicating that inferred population structure is not driven by a small number of closely related accessions.

### 3.3 Migration signals, genetic diversity, and population differentiation

**Figure 2:**
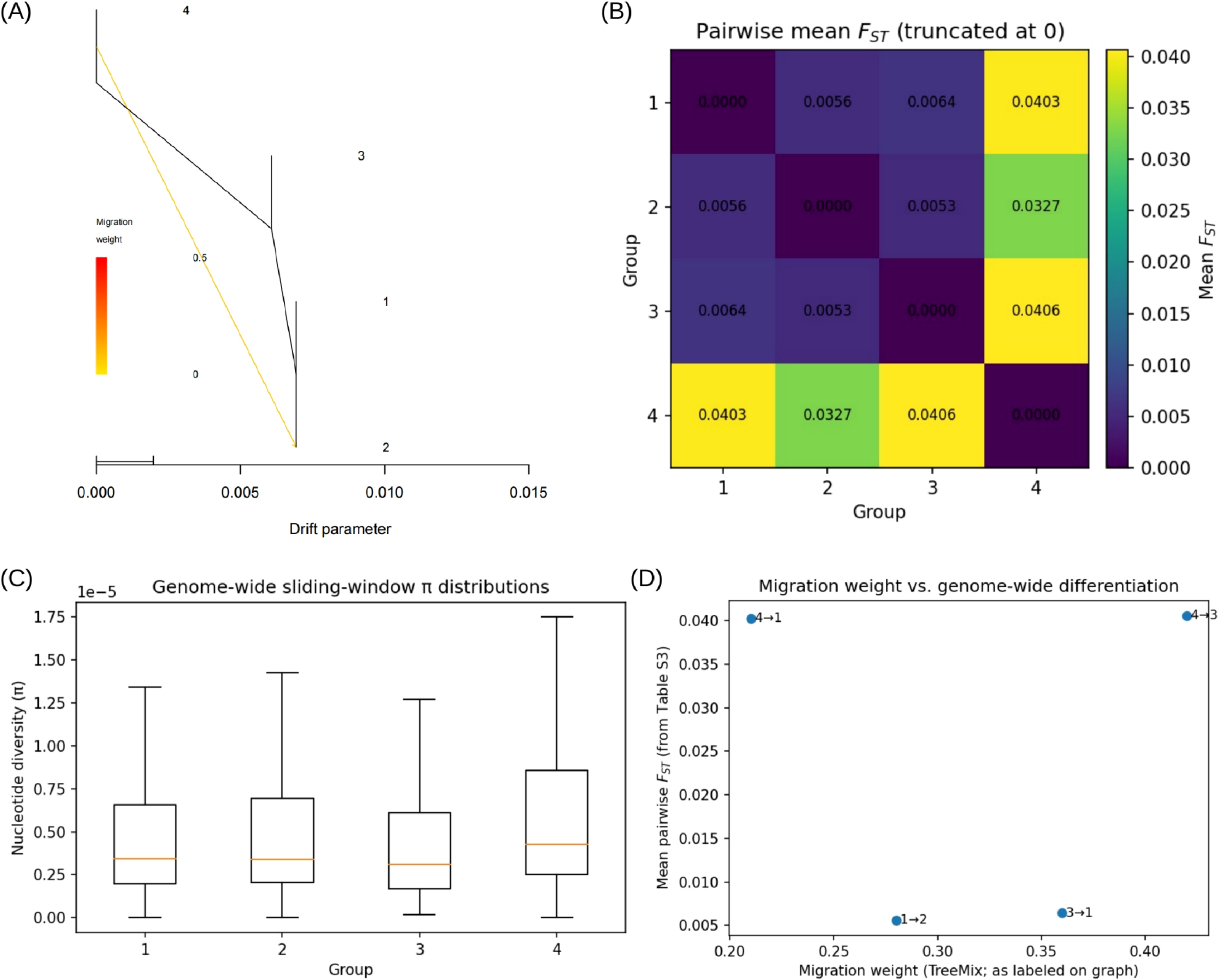
Historical gene flow and genome-wide differentiation among four longan genetic groups. **(A)** TreeMix population graph inferred from genome-wide allele frequency data, showing migration edges among groups 1–4; migration weights are indicated on edges. **(B)** Heatmap of pairwise mean FST among groups 1–4 calculated from sliding-window estimates across the genome (negative values truncated to 0 before averaging). **(C)** Boxplots of sliding-window nucleotide diversity (π) distributions for groups 1–4 across the genome. **(D)** Scatter plot of TreeMix migration weights (as labeled in A) versus corresponding pairwise mean FST values, with points annotated by donor→recipient direction.

To explore possible historical gene flow among the four inferred groups, we applied TreeMix to group-level allele frequency data. The fitted graph suggested several non-tree-like relationships among groups (**Figure 2A**), with the largest inferred migration edge connecting group 4 and group 3. Additional edges connected groups 3 and 1, 1 and 2, and 4 and 1. Because TreeMix-based migration inferences can be sensitive to model settings and because residual diagnostics from alternative edge numbers were not retained, these edges are interpreted here as hypothesis-generating patterns rather than definitive demographic events.

Genome-wide diversity and differentiation estimates revealed heterogeneous genetic variation among groups (**Supplementary Table 2**). Genome-wide mean π ranged from 6.44 × 10⁻^6^ in group 3 to 8.99 × 10⁻^6^ in group 4, with intermediate values for group 1 (7.23 × 10⁻^6^) and group 2 (7.32 × 10⁻^6^) (**Supplementary Table 2**; **Figure 2C**). Pairwise differentiation (mean FST, truncated at 0) was low overall, with the highest values observed for contrasts involving group 4: group 3 vs. group 4 (FST = 0.04059) and group 1 vs. group 4 (FST = 0.04027), followed by group 2 vs. group 4 (FST = 0.03267). Other contrasts were substantially lower (group 1 vs. group 3: 0.00643; group 1 vs. group 2: 0.00559; group 3 vs. group 2: 0.00531) (**Supplementary Table 2**; **Figure 2B**). These results indicate little to low genome-wide differentiation among groups, with comparatively higher divergence consistently involving group 4.

### 3.4 Linkage disequilibrium decay and genomic regions with putative selection signatures

**Figure 3:**
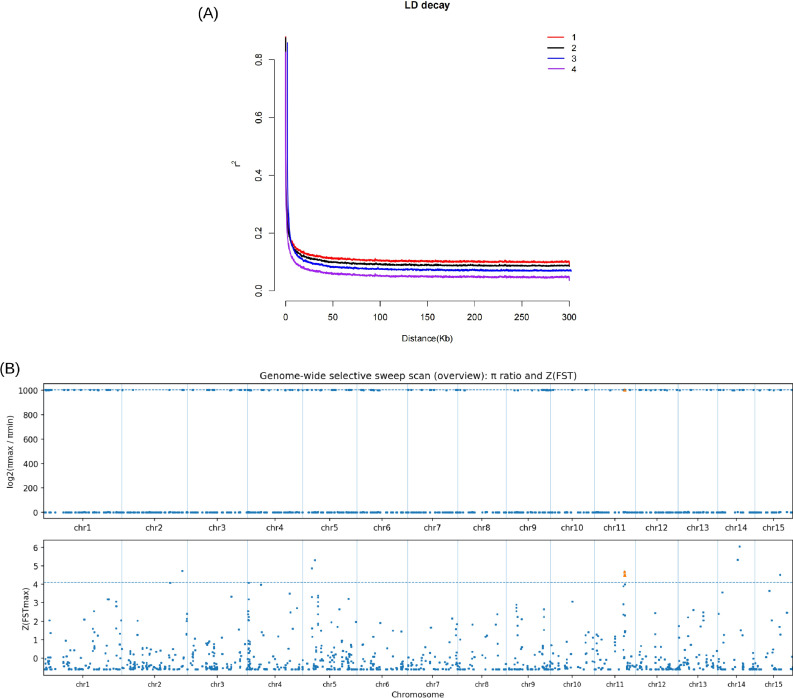
Linkage disequilibrium decay and genome-wide scan for putative selective sweeps. **(A)** Genome-wide LD decay across four longan genetic groups (1–4), estimated by plotting mean pairwise linkage disequilibrium (r²) against physical distance between SNPs (Kb). Curves were calculated from genome-wide SNP pairs and summarize LD decay with increasing distance for each group. **(B)** Genome-wide scan for putative selective sweeps using sliding-window statistics derived from genome-wide SNPs. The upper panel shows log2(πmax/πmin) for each window, where πmax and πmin are the maximum and minimum nucleotide diversity values among groups 1–4. The lower panel shows Z(FSTmax), the Z-score–standardized maximum pairwise FST among the six group comparisons for each window. Points are plotted at window midpoints along the cumulative genomic coordinate, vertical lines indicate chromosome boundaries, dashed horizontal lines denote the top 1% thresholds, and windows exceeding both thresholds are highlighted as candidate selective-sweep intervals.

Linkage disequilibrium (LD) decay was analyzed to assess historical recombination and breeding history across the four longan groups. LD decay curves showed broadly similar decay trends among groups with detectable differences in decay rate (**Figure 3A**). Overall, LD declined with increasing physical distance, consistent with erosion of long-range LD by historical recombination in a long-lived perennial species.

To identify genomic intervals potentially shaped by long-term selection, we scanned the genome using sliding-window nucleotide diversity (π) and pairwise genetic differentiation (FST) calculated from genome-wide SNPs (**Supplementary Table 2**; **Figure 3B**). The scan comprised 7,000 windows of 100 kb with a 10 kb step. For each window, we computed log2(πmax/πmin) across the four groups and FSTmax (the maximum among six pairwise FST comparisons), with FSTmax further standardized as Z(FSTmax). Genome-wide, FSTmax was generally low (median 0.0168), whereas the top 1% threshold corresponded to FSTmax ≥ 0.5102 (equivalent to Z(FSTmax) ≥ 4.096).

Using the joint criterion of exceeding the top 1% thresholds for both metrics, we identified 10 candidate windows showing concurrent diversity depletion and high differentiation (**Figure 3B**). All 10 windows clustered within a single interval on chr11 (17.79–17.98 Mb; ~0.19 Mb). Within this region, group 2 exhibited π = 0 across all 10 windows, while the other groups retained measurable diversity (group 1: 2.21–3.68 × 10⁻^6^; group 3: 0.98–1.43 × 10⁻^6^; group 4: 0.70–1.10 × 10⁻^5^). Differentiation in the same interval was consistently extreme, with the highest FST always observed between group 2 and group 4 (FST_2_;vs_4_ = 0.553–0.575), exceeding the genome-wide 99th percentile cutoff. This interval was prioritized as a candidate region for follow-up analyses. Because the selection scan was summarized in chromosome coordinates whereas the available annotation resource for this revision was scaffold-based, the candidate interval could not be mapped unambiguously to gene models. Therefore, candidate-gene extraction and GO/KEGG enrichment were not performed in this study.

## 4 Discussion

### 4.1 Population structure of longan germplasm and its likely historical drivers

Using dense genome-wide SNPs, our study consistently resolved the 185 longan accessions into four genetic groups across NJ, PCA, and ADMIXTURE analyses. This concordance indicates that the observed stratification reflects robust genome-wide structure rather than a method-specific artifact (Alexander et al., 2009; Patterson et al., 2006). Importantly, these four groups should not be viewed as fully isolated or strictly geography-exclusive lineages. Instead, they are better interpreted as partially differentiated germplasm components within a cultivation system shaped by regional maintenance, clonal propagation, and repeated human-mediated exchange of elite materials.

When considered together with the accession-origin metadata in **Supplementary Table 1**, the inferred groups show broad but incomplete correspondence with sample provenance. Such a pattern is biologically plausible for longan: cultivars are often maintained locally for long periods, which preserves regional structure, but they are also frequently introduced across production areas, which weakens strict geographic partitioning. The presence of admixed ancestry in multiple accessions further supports the view that longan germplasm history is characterized by both regional differentiation and subsequent inter-regional movement. Similar complexity has been reported in a close Sapindaceae relative, lychee, where genome-enabled analyses revealed structured diversity shaped by domestication and subsequent cross-region exchange rather than a simple bifurcating history (Hu et al., 2022).

Relative to previous longan genomic studies, the present work contributes an integrated view of structure, relatedness, differentiation, and LD features within a single resequenced germplasm panel. This type of harmonized population-genomic baseline extends the genomic resources established by earlier longan genome studies and provides a practical framework for germplasm curation and breeding-oriented follow-up (Lin et al., 2017; Wang et al., 2022; Zhao et al., 2026).

### 4.2 Diversity and differentiation: implications for germplasm conservation and utilization

Genome-wide nucleotide diversity differed among the four inferred groups, indicating that standing genetic variation is unevenly distributed across the longan germplasm panel. Such heterogeneity is consistent with perennial crop systems in which long-term local maintenance, clonal propagation, founder effects, and region-specific selection can differentially preserve or erode diversity. In contrast, pairwise differentiation remained generally weak, with all mean FST values below 0.05, indicating little to low genome-wide differentiation despite the clear four-group structure resolved by NJ, PCA, and ADMIXTURE. Together, these results suggest that longan germplasm is best viewed not as a set of deeply diverged lineages, but as a partially structured yet historically interconnected gene pool shaped by both regional differentiation and repeated germplasm exchange. Similar complexity has been reported in other perennial fruit crops, including lychee, where structured diversity coexists with signals of historical introgression and exchange (Hu et al., 2022).

From an applied perspective, the coexistence of four groups together with heterogeneous diversity suggests that these groups should not be treated as interchangeable reservoirs of allelic variation. For conservation, sampling strategies that include representatives from all groups are more likely to maximize retained diversity while reducing the risk of over-representing closely related materials. For breeding and pre-breeding, structure-aware parent selection may help broaden genetic backgrounds and improve the efficiency of combining useful alleles from partially differentiated ancestry groups. In this context, the present study extends earlier longan genomic resources by integrating structure, relatedness, diversity, differentiation, and LD information within a single resequenced germplasm panel, thereby providing a more practical framework for germplasm curation and breeding-oriented utilization (Lin et al., 2017; Wang et al., 2022; Zhao et al., 2026).

### 4.3 Gene-flow hypotheses from TreeMix and cautions in interpretation

TreeMix provides a complementary view of population history by allowing migration edges superimposed on a population tree, modeling non-tree-like relationships from allele-frequency covariance (Pickrell and Pritchard, 2012). In our panel, TreeMix inferred asymmetric migration edges, with the strongest edge involving group 4 as a donor. While these edges offer plausible hypotheses consistent with long-term germplasm movement, admixture-graph inferences can be sensitive to model specification (e.g., the number of migration edges, block size, and residual fit). Therefore, we interpret TreeMix results conservatively, emphasizing cross-method consistency rather than treating migration edges as definitive demographic reconstructions.

Notably, inferred gene-flow patterns are qualitatively concordant with other lines of evidence: accessions showing mixed ancestry in ADMIXTURE are expected under historical exchange and introgression, and the group most frequently involved in TreeMix edges also shows comparatively higher divergence in several pairwise contrasts. Taken together, these findings support a non-uniform history shaped by both divergence and exchange, which is biologically plausible for perennial fruit crops where cultivars are frequently introduced, selected locally, and redistributed across regions. More formal demographic modeling and diagnostic outputs (e.g., residual-fit analyses and robustness across multiple TreeMix settings) would further strengthen inference and represent an important direction for future work.

### 4.4 LD decay, kinship, and selection-scan candidates: interpretation and limitations

LD decay reflects the combined effects of recombination, demography, and selection on haplotype structure. In long-lived perennial crops, LD patterns can additionally be shaped by clonal propagation, uneven breeding intensity, and region-specific maintenance histories. The broadly similar LD decay trends observed among the four groups, together with detectable differences in decay rate, are therefore consistent with partially shared but non-identical demographic and improvement histories across the longan germplasm panel (Chang et al., 2015; Lin et al., 2017; Wang et al., 2022; Zhao et al., 2026).

Kinship analysis provides an additional layer of context for interpreting germplasm structure. The overall kinship distribution centered near zero indicates that most accession pairs are distantly related, whereas the long tail and the small number of highly related pairs likely reflect clonal propagation, duplicate or synonymous accessions, or closely related selections maintained in orchard and breeding systems. Importantly, the major population-structure patterns were not altered by sensitivity checks excluding representative high-kinship pairs, supporting the robustness of the inferred four-group structure (Yang et al., 2011).

Finally, the integrated diversity–differentiation scan identified a narrow interval on chr11 that showed both extreme local differentiation and marked diversity depletion, especially in group 2. This pattern makes the interval a strong priority for follow-up because it is unlikely to reflect random genome-wide background variation alone. At the same time, we avoid over-interpreting this region as a confirmed domestication or breeding locus. A robust biological interpretation will require coordinate-consistent gene models so that the chromosome-based interval can be matched unambiguously to annotated genes. Because such annotation compatibility was not available for the present analysis, we treat the chr11 signal as a prioritized population-genomic target rather than a functionally resolved locus. Future work integrating a matched chromosome-level genome annotation, trait data, and breeding-history information will be required to determine whether this interval is associated with agronomic, developmental, or adaptation-related variation in longan (Weir and Cockerham, 1984; Danecek et al., 2011; Lin et al., 2017; Wang et al., 2022; Zhao et al., 2026).

## 5 Conclusion

In this study, we generated a genome-wide SNP resource from whole-genome resequencing of 185 longan accessions and used it to characterize population structure, genetic diversity, and candidate signals of selection. Multiple complementary analyses consistently resolved the germplasm panel into four genetic groups, with evidence of admixture among subsets of accessions, indicating a history shaped by both divergence and exchange. Genome-wide diversity and differentiation metrics revealed heterogeneous standing variation among groups and generally low overall differentiation, while LD decay patterns further suggested non-uniform demographic and breeding histories. By integrating sliding-window diversity and differentiation scans, we identified a localized candidate interval on chr11 (17.79–17.98 Mb) showing concurrent diversity depletion and elevated differentiation, thereby providing a prioritized genomic region for future validation and functional follow-up. Collectively, these results establish a population-genomic baseline for longan germplasm conservation, curation, and breeding-oriented utilization.

## Data Availability

The original contributions presented in the study are included in the article/supplementary material. Further inquiries can be directed to the corresponding authors.
